# Availability and Quality of Coronary Heart Disease Family History in Primary Care Medical Records: Implications for Cardiovascular Risk Assessment

**DOI:** 10.1371/journal.pone.0081998

**Published:** 2014-01-09

**Authors:** Paula Dhiman, Joe Kai, Laura Horsfall, Kate Walters, Nadeem Qureshi

**Affiliations:** 1 School of Medicine, Division of Primary Care, University of Nottingham, University Park, Nottingham, United Kingdom; 2 The Research Department of Primary Care and Population Health, University College of London (UCL), London, United Kingdom; Charité University Medicine Berlin, Germany

## Abstract

**Background:**

The potential to use data on family history of premature disease to assess disease risk is increasingly recognised, particularly in scoring risk for coronary heart disease (CHD). However the quality of family health information in primary care records is unclear.

**Aim:**

To assess the availability and quality of family history of CHD documented in electronic primary care records

**Design:**

Cross-sectional study

**Setting:**

537 UK family practices contributing to The Health Improvement Network database.

**Method:**

Data were obtained from patients aged 20 years or more, registered with their current practice between 1^st^ January 1998 and 31^st^ December 2008, for at least one year. The availability and quality of recorded CHD family history was assessed using multilevel logistic and ordinal logistic regression respectively.

**Results:**

In a cross-section of 1,504,535 patients, 19% had a positive or negative family history of CHD recorded. Multilevel logistic regression showed patients aged 50–59 had higher odds of having their family history recorded compared to those aged 20–29 (OR:1.23 (1.21 to 1.25)), however most deprived patients had lower odds compared to those least deprived (OR: 0.86 (0.85 to 0.88)). Of the 140,058 patients with a positive family history recorded (9% of total cohort), age of onset was available in 45%; with data specifying both age of onset and relative affected available in only 11% of records. Multilevel ordinal logistic regression confirmed no statistical association between the quality of family history recording and age, gender, deprivation and year of registration.

**Conclusion:**

Family history of CHD is documented in a small proportion of primary care records; and where positive family history is documented the details are insufficient to assess familial risk or populate cardiovascular risk assessment tools. Data capture needs to be improved particularly for more disadvantaged patients who may be most likely to benefit from CHD risk assessment.

## Introduction

Family history has long been recognised as an important risk factor for a range of common chronic diseases, such as cancers, diabetes, and coronary heart disease (CHD) [1], [2]. The recognised predictive accuracy of family history of CHD has opened international interest in integrating family history information into routine clinical practice [3]–[5]. Currently, several risk prediction models incorporate family history. In the UK, these include cardiovascular risk assessment tools such as ASSIGN [6] and QRISK2 [7]. With the recognition of CHD as the leading cause of death, such assessment has been implemented through national guidance [8] and policy initiatives [9].

In many primary care computer systems, automated risk prediction tools can be populated from coded data in clinical records and provide an ideal environment to collect and assess patient CHD risk. In primary care, the initial consultation and/or health screening questionnaire at new registration is a well-recognised opportunity to collect family history information [10]–[12]; and the 1990 UK Family Practice contract has encouraged enquiry about inherited predisposition [13].

Data extracted from primary care computer systems have previously been used to identify the extent of missing data for standard cardiovascular risk factors [14]. A similar approach could be used to explore the nature of the family history information recorded. The potential value of electronic health records to assimilate important information on family history has been recognised [15].

Level of recording of family history has been collated for cancer and CHD in primary care-based cross-sectional surveys and in community-based epidemiological studies [16], [17]. Similarly, web based tools have been developed to collate this information [18]. Thus far, quality of family history information has not been evaluated in primary care databases.

The quality of data entry in primary care computer systems has been evaluated for medication recording [19], but is yet to be assessed for family history. Accurate assessment of the relationship of the affected relative to the patient, and the age of onset of the condition will indicate quality of data recording necessary to assess the CHD risk associated with a family history [20], [21].

This study aimed to assess the availability and quality of family history of CHD data documented in electronic primary care records at patient registration, and any associations with patients' socio-economic background.

## Materials and Methods

### Design and Setting

This was a cross-sectional study, using data from The Health Improvement Network (THIN) database, a large anonymised primary care database. At the time the study was conducted, the THIN database contained data from 537 practices, on 10.9 million patients, representing approximately 6% of the UK primary care population. Data in this database has a similar distribution to the UK population. Data is entered using the VISION family practice computer software, by the family physician, nurses and administrative staff during routine clinical practice; and is recorded using a hierarchal coding system (Read Codes), which maps onto the ICD-10 coding system, and free text entries. As well as medical diagnoses and symptoms, the THIN database also includes information on prescriptions, referrals, tests and risk factors relating to lifestyle behaviour, and is linked to UK Census data on local area deprivation. Recording of consultations and prescriptions is comparable to national consultation and prescription statistics [22]. Disease rates have been calculated from the data and compared to externally generated rates in validation studies [23].

### Study Population

Eligible patients were aged 20 or greater, and registered with their practice during the decade 1^st^ January 1998 to 31^st^ December 2008 for at least one year. Patients without gender specified or patients registered before the practice began using the VISION medical record software were excluded. Temporary patients are not formally registered with UK Family Practice and thus data input is suboptimal in these patients. In line with established methodology in primary care database research, to avoid the artificial inflation of the denominator with temporary patients, patients who were not registered with the practice for at least one year were removed from the cohort. To identify the level of family history recording, the cohort comprised new patient registrants with any family history of CHD. To assess quality of data, only patients who had presence of a positive FH of CHD recorded were included.

### Outcomes

Code lists for family history (FH) were created using recognised primary care database methodology [24] which identified FH recording in the medical records and additional health detail records. Code lists used are available from authors on request. Anonymised and coded free text was also investigated to derive information on specific first and second degree relatives and age of onset.

For recording of CHD the outcome measure was a dichotomised variable indicating whether any FH (positive or negative) of CHD was recorded or not. A positive FH of CHD would encompass any description indicating information on FH of CHD was present, whilst a coded entry ‘no family history of CHD’ was classified as a negative family history.

The outcome for the quality of data was restricted to those with a positive FH of CHD. It was then sub-classified into four categories: FH with no further details identified; FH with relative identified; FH with age of onset identified; and FH with details on both relative and age of onset.

### Covariates

For both outcomes the patient's gender, age (10 year age groups), level of deprivation and year of practice registration (1^st^ Jan 1999–31^st^ Dec 2008) were included as covariates in our study. The level of deprivation was defined using the Townsend Index, derived from the patient's postcode and combining 4 census variables: car ownership, household size, owner occupation, employment status [25]. Each postal enumeration district (around 150 households) is assigned a Townsend Deprivation score. These are divided into national quintiles and in the THIN database patients are assigned a quintile score according to the enumeration district in which they live. This score ranges from 1–5, where a higher score indicates greater deprivation.

### Statistical Analyses

Initial descriptive presentation of the covariates included numbers (%) for categorical variables; mean (SD) for normally distributed variables; and median (IQR) for non – normally distributed variables. Associations between groups (categorical data, such as gender) were tested for, using Chi-squared tests, whilst for non-normal ordinal continuous data (such as Townsend Score), Kruskal Wallis tests were used.

Due to the hierarchical structure of the data where patients were clustered by practice, a multilevel approach was adopted for the analyses of both objectives. A two-level model was used, allowing for a random intercept by the cluster variable (practice). An unadjusted and adjusted analysis was performed. The latter adjusted for all the covariates in the study.

A multilevel logistic regression analysis was used to assess recording of FH of CHD data, odds ratios indicating the odds of having a FH recorded at practice registration versus not having FH recorded. Whilst the quality of family history data was assessed by performing a multilevel ordinal logistic regression analysis. For this analysis having family history recordings with both items (age of onset and relatives affected) was given highest quality ranking, whilst having a single item (age of onset or relative) were considered of equal quality. The poorest quality was recording FH of CHD with no further details specified. Results from this analysis gave the odds of having the best FH quality (FH of CHD with relative and age specified) versus the two lower qualities combined (FH of CHD with age or relative given, and FH of CHD alone).

Analysis also estimated practice variation, presented as a value of the variance and standard error at level 2 (practice) and also a percentage of the remaining unexplained variance of the final model, using the variation partition coefficient (VPC). To calculate the VPC, the variance at level 1 (patient level) is assumed to be 3.29 (the variance of a standard logistic regression). As the models produced are two level random intercept models the VPC can also be interpreted as the Intra Class Correlation (ICC), the amount of variance attributable to practices. Missing values were presented in the descriptive analysis, but were excluded from the logistic regression analysis. Analyses were performed using Stata v11 [26] and MLwiN v2.23 statistical software [27].

### Ethics statement

The Health Improvement Network (THIN) scheme of providing anonymised data to researchers was approved by the NHS South-East Multi-centre Research Ethics Committee and the CSD Medical Research Scientific Review Committee approved the present study.

## Results

### Recording of family history of CHD

Of the total cohort, 283,715 (18.86%) patients had FH of CHD recorded (positive or negative); half (49.37%) of these had a positive FH of CHD recorded, representing 9.31% of the total cohort. Socio-demographic characteristics of the sample are provided in Table 1.

**Table 1:** Demographic characteristics of patients in the complete sample.

Variable		Total (N = 1,504,535)*		Patients with any FH of CHD recorded (n = 283,715)**		Patients with positive FH of CHD recorded (n = 140,058)***	
N (%)							
Gender	Male	703,498	46.8%	132,151	46.6%	62,825	44.9%
Age (years)****	20–29	550,611	36.6%	96,225	33.9%	36,577	26.1%
	30–39	395,708	26.3%	77,306	27.3%	37,204	26.6%
	40–49	205,697	13.7%	44,061	15.5%	26,634	19.0%
	50–59	134,696	9.0%	31,801	11.2%	20,645	14.7%
	60–69	85,901	5.7%	19,726	7.0%	12,062	8.6%
	70–79	55,845	3.7%	9,677	3.4%	5,144	3.7%
	80 and above	61,610	4.1%	4,866	1.7%	1,767	1.3%
	Missing	14,467	1.0%	53	0.0%	25	0.0%
Townsend Score *****	1	303,573	20.2%	55,767	19.7%	29,951	21.4%
	2	275,432	18.3%	54,259	19.1%	28,009	20.0%
	3	302,589	20.1%	56,361	19.9%	27,437	19.6%
	4	299,197	19.9%	56,562	19.9%	25,947	18.2%
	5	222,960	14.8%	40,761	14.4%	17,889	12.8%
	Missing	100,784	6.7%	20,005	7.1%	10,825	7.7%
Median [IQR]							
Year of Registration		2004 [2002–2007]		2004 [2002–2007]		2005 [2002–2007]	
Age (years)		30–39 [20–29, 40–49]		30–39 [20–29, 40–49]		30–39 [20–29, 50–59]	
Townsend Score		3 [2–4]		3 [2–4]		3 [2–4]	

Percentages are proportions of the total cohort (1,504,535).

Percentages are proportions of the cohort of patients with any FH recorded (283,715).

Percentages are proportions of the cohort of patients with positive FH of CHD recorded (140,058).

Age was missing for 0.96% of the total sample.

Townsend Score was missing for 6.70% of the total sample.

Compared to the total cohort, proportionally fewer men had any (positive or negative) recording of FH of CHD (46.76%, p = 0.16), or of a positive FH of CHD (44.87%). Further, patients with a recorded FH were slightly older (p<0.001), more recently registered with the practice (p<0.001) and had lower deprivation scores (p = 0.007).

**Figure 1:**
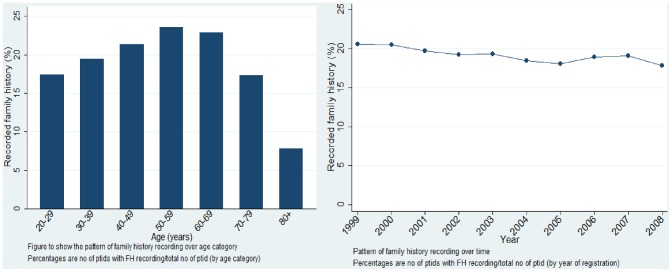
Demographic trends of patients with FH of CHD recorded, by age category and year of registration. Values are out of the total sample: 1,389,965 (excl missing values). Reports the proportion of patients with any FH of CHD within each subgroup (age group and year of practice registration).

The proportion of patients with any FH of CHD recorded reduced over time from 20.55% of patients with a record of FH of CHD in 1999 to 17.82% in 2008. The most prevalent recording of any FH was in the age groups 50–59 and 60–69 (Figure 1). This pattern remained the same when exploring FH recording for each age group by year of registration. Patients aged 80 and above had a consistently low recording of FH. On the other hand, no pattern was found between the level of recording and patient deprivation across different years of registration, for example in 2007 the highest level of recording was for those patients in Townsend Score 5, and in 2003 & 1999 the highest score was for patients with Townsend Score 2. Multivariate analysis confirmed that the level of recording fell in patients aged over 70 (OR: 0.66 (0.64 to 0.68)) and with increasing deprivation score (Table 2). For example, patients with a Townsend Score of 5 (most deprived) had OR: 0.86 (95%CI: 0.85 to 0.88) of having their FH recorded compared to patients with a Townsend score of 1 (least deprived). Further, women were slightly more likely to have a FH recording (OR: 1.07 (95%CI: 1.06 to 1.08).

**Table 2:** Unadjusted and adjusted odds ratios (95% CI) for recording of FH of CHD, from the multilevel logistic regression model*.

Variable		Unadjusted Analysis	Adjusted Analysis**
Sex	Male	Reference	
	Female	1.02 (1.01 to 1.03)	1.07 (1.06 to 1.08)
Age (years)	20–29	Reference	
	30–39	1.11 (1.10 to 1.12)	1.11 (1.10 to 1.13)
	40–49	1.16 (1.15 to 1.18)	1.17 (1.15 to 1.19)
	50–59	1.23 (1.21 to 1.25)	1.23 (1.21 to 1.25)
	60–69	1.08 (1.05 to 1.10)	1.07 (1.05 to 1.09)
	70–79	0.66 (0.64 to 0.68)	0.66 (0.64 to 0.68)
	80+	0.24 (0.24 to 0.25)	0.24 (0.23 to 0.25)
Townsend Score	1	Reference	
	2	0.96 (0.95 to 0.98)	0.97 (0.95 to 0.98)
	3	0.93 (0.91 to 0.94)	0.94 (0.92 to 0.95)
	4	0.91 (0.90 to 0.93)	0.92 (0.91 to 0.94)
	5	0.86 (0.84 to 0.87)	0.86 (0.85 to 0.88)
Period	1999	1.10 (1.07 to 1.13)	1.09 (1.06 to 1.12)
	2000	1.08 (1.05 to 1.10)	1.07 (1.04 to 1.09)
	2001	1.09 (1.06 to 1.11)	1.08 (1.06 to 1.10)
	2002	1.09 (1.06 to 1.11)	1.08 (1.06 to 1.10)
	2003	1.08 (1.05 to 1.10)	1.07 (1.05 to 1.09)
	2004	1.04 (1.01 to 1.06)	1.03 (1.01 to 1.05)
	2005	0.99 (0.97 to 1.01)	0.99 (0.97 to 1.01)
	2006	1.10 (1.08 to 1.12)	1.09 (1.07 to 1.11)
	2007	1.11 (1.09 to 1.13)	1.11 (1.09 to 1.13)
	2008	Reference	

Results are derived using 2^nd^ order linearisation and PQL estimation type.

Adjusted for sex, age category, year of registration and Townsend score.

Multivariate analysis also demonstrated marginally better recording of FH in years prior to 2008, but no specific trend with time was found. Analysis indicated that after adjusting for gender; age; deprivation; and year of registration, 45.26% (VPC: 2.72 (SE: 0.13)) of the unexplained variance of having FH of CHD recorded is due to between practice variation.

### Quality of family history recording in primary care computer records

Of the 1,504,535 total cohort, 140,058 (9.31%) had presence of a positive FH of CHD recorded. Of these, 33.34% (95% CI: 33.09% to 33.59%) had a FH of CHD recorded with no further information; 10.77% (95% CI: 10.61% to 10.93%) had FH of CHD recorded with information on the affected relative; 45.05% (95% CI: 44.78% to 45.30%) had FH of CHD recorded with the age of onset specified; and 10.85% (95% CI: 10.69% to 11.01%) had FH of CHD recorded with both the relative and age of onset specified.

**Figure 2:**
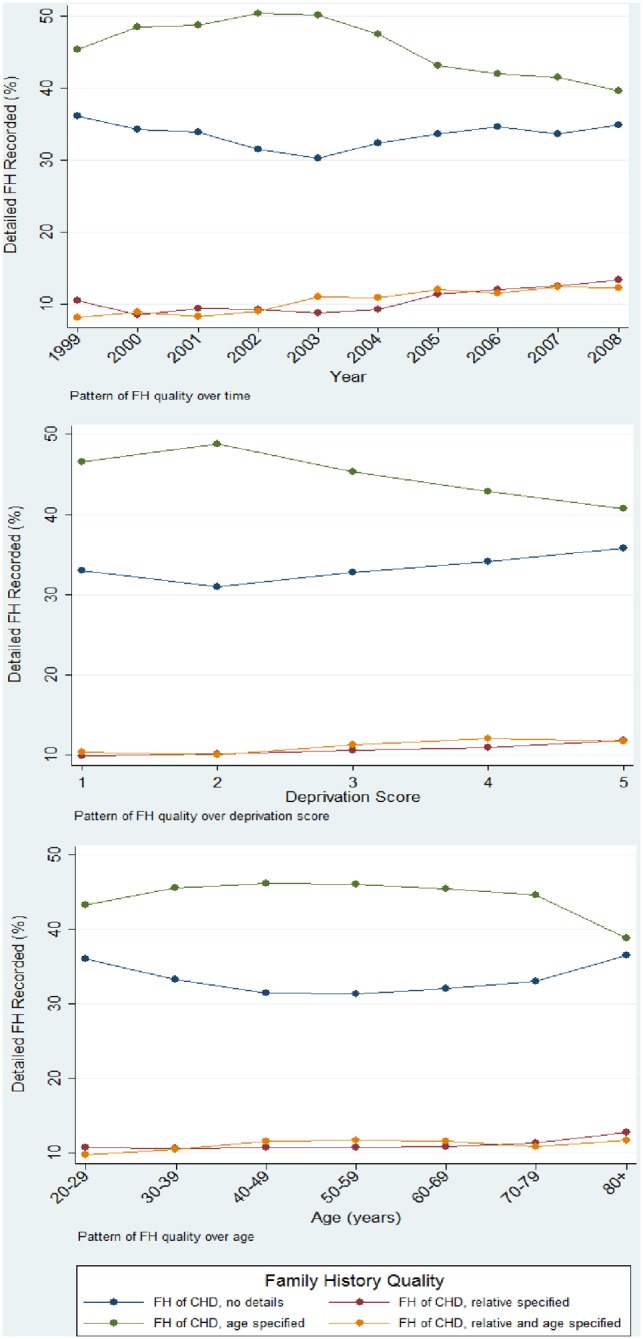
Pattern of FH quality by year of registration, Townsend score and age category (excluding missing values).

When analysed in a multilevel adjusted ordinal logistic regression, (collapsing FH categories, age of onset category and relative affected, into a single category) there was no association between quality of FH and the covariates: year of registration, deprivation score and patient's age. Further, as demonstrated in Figure 2, across all of the covariates the highest availability of FH recording was where no further details are specified or age of onset is also identified with the FH; with poor levels of recording in other categories (relative noted, and both relative and age of onset noted).

The unexplained variation at practice level for the final model of the quality of FH recording is 50.67% (VPC: 3.38 (SE: 0.10)), after controlling for gender, age, deprivation and year of registration.

## Discussion

### Summary of main findings

We have found CHD family history recording in UK primary care is low, and, where a positive history is recorded, it often lacks details to adequately assess familial risk. Only 19% of patients at 537 practices (with in excess of 3 million actively registered patients) had the presence of any family history of CHD recorded, in the decade to 2008. Presence of a positive family history of CHD was noted in 9% of patients, but data combining age of onset and specified relative affected was available in only 11% of these records.

### Strengths and limitations

To our knowledge, this is the first study to also explore nature of the family history in primary care computer records, providing a comprehensive cross-section of their status from 1998 to 2008 in the UK.

The THIN database is derived from 537 practices and contains data on 6.2% of the UK population. Data from this database is generalisable to the UK demographic for age and gender distribution. Crude prevalence rates for common chronic diseases in THIN are also similar to national estimates; for example, the crude prevalence of coronary heart disease in THIN was 3.9% compared to 3.7% in the UK national QOF data [28].

However the THIN database contains slightly more affluent patients (23.5% classified least deprived compared to 20% nationally). This may have caused some overestimation of the total proportions we found with FH recorded, but differences are marginal. Further, the THIN database contains fewer patients aged less than 25 years. This does not negate the relationship between age and extent of family history recording. A further limitation of the THIN database is that the Townsend Deprivation Score is not recorded individually, but is based on the patient's residential postcode (the approximately 150 households in the surrounding neighbourhood) and this deprivation status may not reflect that of the individual.

Although having a detailed family history recorded does not imply an accurate one, previous studies have reported the accuracy of a recorded family history of CHD, compared to direct recall by relatives, is as high as 67% [29]. Further, we anticipate that family history recording would be more frequently taken at registration, compared to other clinical encounters [11]. We also attempted to identify free text entries on family history in records, but recognise potentially relevant information to assess its quality may not have been identified, such as, free text on exact relative affected.

Considering the analysis, missing values were handled using a complete case analysis. As a result 8% of the total sample was not included in the analysis; Townsend score was not available for approximately 7% of the sample; and 1% for age. Patients without Townsend score recorded had similar distributions for gender and age; but registered with their practice later. Patients with age recorded had similar distributions for deprivation and year of registration; but had a higher proportion of males compared to the total cohort. The slightly different profile for those with missing values should not impact on the overall study findings.

### Existing literature

Secondary findings of similar database studies have shown FH of CHD recording to range between 3.7% [30] and 10.8% [31] of patients, and specifically between 3.7% [30] and 4.4% [32] for studies using the THIN database.

These estimates are significantly lower than our level of recording of 19%, which included patients identified with FH of CHD based on free text extracted from electronic health records. Compared to the low level of recording of family history in primary care records, epidemiological research indicates up to 52% of the population will have a positive family history of CHD, whilst 37% will have a FH of premature CHD [17]. However, similar to this study, research in secondary care indicate poor recording of family history [33].

Case reviews in other countries, looking at small numbers of primary care practices have also reported poor recording of FH in electronic health records. A Swedish study reviewing cardiovascular risk factors in hypertensive patients, showed family history was incompletely recorded (46%) in patient records [34]. Similar studies in New Zealand have indicated varied recording of cardiovascular risk factors. High levels of recording was found of some risk factors such as blood pressure and cholesterol and smoking [35], but levels of FH recording ranged from 19 to 81% in patients aged 60–75 and was dependent on the practice [36].

In the current study, better recording was anticipated in newly registered patients due to family physicians and practice nurses recognising the value of recording family history on registration [10], [11], however the level and quality of recording was still low.

### Implications for clinical practice

Availability of family history data was greatest in those aged 50 to 69 years, and was lower in the more socially deprived. Yet recognition of family history of premature CHD in younger age groups and in socially deprived contexts is likely of greater value in targeting interventions to patients with greatest overall risk of premature cardiovascular disease events [6]. This should form a priority for practice.

Optimal interpretation of familial risk requires both age of diagnosis and relative affected [21], [37] but this was available in only one in ten patients identified as having a family history of CHD. Poor recording of details on affected relatives may reflect limited choice of history-taking codes in primary care computer systems and the time required in consultations. Currently, electronic health records used in UK primary care lack integrated family history pedigree software, with clinicians relying on unstructured formats to enter FH data [38]. The extent of missing data on affected relatives highlights the possible limitations of risk prediction tools that incorporate family history information from clinical computer medical records. Thus, this study underlines the need to introduce more structured approaches to allow clinicians to improve capture of family history data in clinical practice.

Paucity of family history data may compromise scoring of cardiovascular risk if this is automatically calculated for patients from routinely collected data in primary care records [30], [37]. For example, in the Joint British Societies CVD risk assessment tool [37], identifying family history of CHD in a male 1st degree relative under 55 and/or female relative under 65 uplifts the CVD risk score by a factor of 1.5. Applying the current study findings, around 85% of patients in primary care (81% no recording & 4% poor recording) would not have sufficient family history data recorded to benefit from this assessment of their cardiovascular risk. Caution is thus also advised when interpreting studies of risk assessment tools that use family history derived from primary care computer records given the current extent of missing and poor quality data.

A recent randomised trial in primary care suggests the addition of more systematic family history may increase the proportion of individuals identified at high cardiovascular risk for targeting of preventive interventions by up to 40% [39]. We anticipated improved recording of CHD family history over the decade from 1998 given the increased prominence of cardiovascular risk assessment tools in primary care [8], but this was not found. There is a danger that continued incomplete collection of family history data will lead to imprecise cardiovascular risk assessment in those at high familial risk. Implementation of population screening, such as England's Vascular Health Check programme and cardiovascular risk assessment in the UK Primary Care Quality Outcome incentivisation scheme, may help [9].

Future research should explore approaches to improving the more systematic recording of family history of CHD and other common chronic diseases in primary care, together with qualitative exploration of barriers and facilitators to family history collection in practice [40]. Feedback and incentives, for example, may improve data quality [41]. This may enable data on this key risk factor to be used with more confidence and for benefit in patient health outcomes.
